# Trends in central nervous system-active polypharmacy among people with multiple sclerosis

**DOI:** 10.1177/13524585241251986

**Published:** 2024-05-15

**Authors:** Hayden Naizer, Joseph Wozny, Trudy Millard Krause, Ethan Huson, Leorah Freeman

**Affiliations:** Department of Neurology, Dell Medical School, The University of Texas at Austin, Austin, TX, USA; McGovern Medical School, The University of Texas Health Science Center at Houston, Houston, TX, USA; Department of Management, Policy, and Community Health, School of Public Health, The University of Texas Health Science Center at Houston, Houston, TX, USA; Department of Management, Policy, and Community Health, School of Public Health, The University of Texas Health Science Center at Houston, Houston, TX, USA; Department of Neurology, Dell Medical School, The University of Texas at Austin, Austin, TX, USA; Department of Neurology, Dell Medical School, The University of Texas at Austin, Austin, TX, USA

**Keywords:** Polypharmacy, pharmacoepidemiology, central nervous system-active polypharmacy, CNS-active polypharmacy, psychotropic polypharmacy, claims analysis

## Abstract

**Background:**

People with multiple sclerosis (pwMS) are at risk of concurrently using multiple central nervous system (CNS)-active drugs, yet the prevalence of CNS-active polypharmacy remains unmeasured in pwMS.

**Objective:**

The objective is to measure the prevalence of CNS-active polypharmacy in pwMS.

**Methods:**

This serial, cross-sectional study measured CNS-active polypharmacy in people with MS in the United States from 2008 to 2021 using insurance claims data. CNS-active polypharmacy was defined as the concurrent prescription of ⩾3 CNS-active drugs for >30 continuous days. CNS-active drugs included antidepressants, antiepileptics, antipsychotics, benzodiazepines, nonbenzodiazepine benzodiazepine receptor agonist hypnotics, opioids, and skeletal muscle relaxants.

**Results:**

The number of subjects included at each time point ranged from 23,917 subjects in 2008 to 55,797 subjects in 2021. In 2021, subjects with CNS-active polypharmacy were more likely to be 46–65 years of age and have CNS-related comorbidities compared to those without CNS-active polypharmacy. From 2008 to 2021, the age-adjusted prevalence of CNS-active polypharmacy among female subjects increased from 19.8% (95% confidence interval (CI) = 19.1–20.4) to 26.4% (95% CI = 25.9–26.8) versus 15.9% (95% CI = 14.8–17.0) to 18.6% (95% CI = 17.9–19.2) in male subjects.

**Conclusion:**

The prevalence of CNS-active polypharmacy has increased among people with MS with a growing disparity by sex.

## Introduction

People with multiple sclerosis (pwMS) often use central nervous system (CNS)-active drugs to treat MS symptoms including pain, spasticity, fatigue, anxiety, and depression.^1–4^ To manage these symptoms, pwMS may concurrently use multiple CNS-active drugs, known as CNS-active polypharmacy. In pwMS, prevalence estimates of anxiety, depression, and pain are 21.9%, 23.7%, and 63%, respectively.^5,6^ Antiepileptics and skeletal muscle relaxants are commonly used to treat neuropathic pain and spasticity in MS; thus, pwMS have multiple risk factors for CNS-active polypharmacy.^1,3,4^

Definitions of CNS-active polypharmacy are commonly adapted from the American Geriatric Society’s Beers criteria’s strong recommendation against the concurrent use of ⩾3 CNS-active drugs, including antipsychotics, antidepressants, antiepileptics, benzodiazepines, nonbenzodiazepine benzodiazepine receptor agonist hypnotics, opioids, and skeletal muscle relaxants.^7–9^ Studies have used this continually updated list of drugs for standardization of CNS-active polypharmacy definitions across populations.^10–13^ Among older adults, CNS-active polypharmacy is associated with falls and cognitive decline,^7,14–18^ both of which have an increased prevalence in pwMS compared to people without MS.^19^ Despite these associations, CNS-active polypharmacy in pwMS has rarely been measured.^20^

To better understand CNS-active polypharmacy in pwMS, we used insurance claims data in a serial cross-sectional design to measure the trends in the prevalence of CNS-active polypharmacy in pwMS from 2008 to 2021.

## Methods

This observational study used Optum’s de-identified Clinformatics Data Mart Database (CDM) in a serial cross-sectional design from 2008 to 2021.^21^ CDM is derived from a database of administrative health claims for members of large commercial and Medicare Advantage health plans across all 50 US states. PwMS in 2021 were included in a subgroup analysis of covariates and prescriber characteristics.

### Study sample

PwMS were identified using a previously validated algorithm for claims data.^22^ Inpatient and outpatient claims included outpatient, emergency department, and professional medical claims with the International Classification of Diseases (ICD)-10 code “G35” or ICD-9 code “340.”

A list of disease-modifying therapies (DMTs) and their associated efficacy in the treatment of MS is included (Supplemental eTable 1). Drugs were identified using National Drug Codes (NDC) and Healthcare Common Procedure Coding System (HCPCS) codes then checked against the Lexicon Multum drug database. Rituximab was excluded from patient identification given its indications outside of MS. Natalizumab and ozanimod claims were excluded from identification if a person had a diagnosis code indicating inflammatory bowel disease or Crohn’s disease, respectively.

A subject was assigned to an index date according to the earliest claim after which at least two other claims occurred within 1 year. PwMS were included if they were at least 18 years of age and had >30 consecutive days of insurance enrollment within the respective year.

### CNS-active polypharmacy definition and measurement

CNS-active polypharmacy was defined as the concurrent use of ⩾3 CNS-active drugs for >30 days.^7,23^ CNS-active drug classes included antidepressants, antiepileptics, antipsychotics, benzodiazepines, nonbenzodiazepine benzodiazepine receptor agonist hypnotics (“z-drugs”), opioids and skeletal muscle relaxants.^7^ CNS-active drugs were identified using the American Hospital Formulary Service’s (AHFS) Pharmacologic-Therapeutic Classification System (Supplemental eTable 2). Use of multiple drugs within and between drug classes contributed to CNS-active polypharmacy.

Chronic CNS-active polypharmacy was measured as the concurrent use of ⩾ 3 CNS-active drugs for ⩾ 180 days within a year. Subchronic CNS-active polypharmacy was measured as the concurrent use of ⩾ 3 CNS-active drugs for > 30 and < 180 days within a year.

Generic drug names were mapped to simplified CNS-active drug names. For example, prescriptions for “codeine” and “acetaminophen with codeine” were categorized as “codeine.” Periods of drug use were measured using the prescription’s fill date and days supplied. Subjects were considered exposed a drug during gaps between prescriptions of the same drug lasting ⩽ 3 days.

### CNS-active drug use by class

The age-adjusted prevalence of each CNS-active drug class was calculated for each polypharmacy category by sex from 2008 to 2021. If a subject was prescribed a CNS-active drug during a period of CNS-active polypharmacy, they contributed to the prevalence of the drug class for the respective year.

### Covariate measurement

Age, sex, and prevalence of CNS-active drug use by drug class were measured for each time point from 2008 to 2021. Sex was reported as female, male, or unknown.

In the subgroup analysis of pwMS in 2021 additional covariates were compared across CNS-active polypharmacy status by age using age-adjusted prevalence ratios. Geographic region was reported as South, West, Midwest, and Northeast based on US Census Region designations. Rurality was assessed using subject’s ZIP data and the 2010 Rural-Urban Community Area Codes from the US Department of Agriculture. Codes for small town and rural areas were categorized as rural.

DMT use was determined if any pharmacy or medical claim with an NDC or HCPCS codes for a DMT was made. The therapeutic efficacy of the first reported DMT in 2021 was reported as the DMT efficacy. The efficacy of each DMT was determined according to previously reviewed categorizations^24^ (Supplemental eTable 1).

A relapse was estimated by the instance of an inpatient or outpatient claim with a primary diagnosis of MS with an associated corticosteroid prescription within 7 days following the claim.^25^ Durable medical equipment (DME) utilization was measured using HCPCS E codes for mobility related DME such as wheelchairs and stimulation devices. Magnetic resonance imaging (MRI) utilization was measured using Current Procedural Terminology codes for brain, spinal cord, and orbit MRIs. Comorbidities were measured using ICD-10-CM codes. The Charlson Comorbidity Index (CCI) was calculated when at least two diagnoses for the same comorbidity were reported more than 30 days apart. CCI was categorized as 0 indicating no comorbidities, 1 to 2 indicating mild comorbidities, 3 to 4 indicating moderate comorbidities, and ⩾ 5 indicating severe comorbidities. CNS-related comorbidities were and reported individually (Supplemental eTable 3).

For each subject, the number of distinct CNS-active drugs used, and the number of distinct prescribers was reported. The unique provider code was mapped to prescriber types such as internal medicine physician, neurologist, and nurse practitioner as reported in the CDM. Internal medicine, family medicine, and pediatric physicians were categorized as primary care. The number of person-days, days with CNS-active polypharmacy, concurrent CNS-active-drugs prescribed, prescribers, and prescriber types were calculated in 2021. The twenty-five most common CNS-active drugs contributing to CNS-active polypharmacy were reported with age and sex-adjusted chronic-to-subchronic prevalence ratios. Finally, the days supplied for each CNS-active drug prescription contributing to CNS-active polypharmacy were reported by prescriber type.

### Statistical analysis

Direct age-adjustment of the prevalence of CNS-active polypharmacy was performed using the included subjects in each year as the reference group for their respective year of study, and 95% confidence intervals (CIs) were reported and graphed. Prevalence ratios of age-adjusted CNS-active polypharmacy between female and male pwMS were estimated using incidence rate ratios from robust Poisson regression models (RPR). Age-adjustment for the prevalence of drug use by drug class was performed using a direct standardization approach. Using incidence rate ratios from robust RPRs, age and sex-adjusted prevalence ratios were reported for the prevalence of specific CNS-active drugs in pwMS with chronic versus subchronic CNS-active polypharmacy.

Count and proportion for categorical variables were reported while mean and standard deviations were reported for continuous variables. All analyses were performed using Stata Statistical Software: Release 18 (StataCorp LLC, College Station, Texas, USA).

## Results

Table 1 presents the total sample sizes for each year from 2008 to 2021. In 2021, 20,259,327 people were represented in the CDM and 55,941 (0.3%) pwMS were identified. Of total, 144 (0.3%) pwMS were excluded, resulting in a final sample size of 55,797 (Supplemental eFigure 1).

**Table 1.** Age-adjusted prevalence estimates of CNS-active polypharmacy by sex, year, and chronicity from 2008 to 2021.

Year	Female						Male						Female-to-male prevalence ratio^a^		Total sample size^b^
	Overall CNS-active polypharmacy^c^		Subchronic CNS-active polypharmacy^d^		Chronic CNS-active polypharmacy^e^		Overall CNS-active polypharmacy^c^		Subchronic CNS-active polypharmacy^d^		Chronic CNS-active polypharmacy^e^				
	%	(95% CI)	%	(95% CI)	%	(95% CI)	%	(95% CI)	%	(95% CI)	%	(95% CI)	PR	(95% CI)	
2008	19.8	(19.1–20.4)	11.4	(10.9–12.0)	8.3	(7.9–8.8)	15.9	(14.8–17.0)	9.3	(8.4–10.2)	6.6	(5.9–7.4)	1.27	(1.18–1.36)	23,917
2009	20.3	(19.7–20.9)	11.1	(10.7–11.6)	9.1	(8.7–9.6)	15.2	(14.2–16.1)	8.6	(7.8–9.3)	6.6	(5.9–7.3)	1.30	(1.22–1.39)	26,099
2010	20.1	(19.5–20.6)	10.6	(10.2–11.1)	9.4	(9.0–9.9)	16.0	(15.1–16.9)	8.9	(8.2–9.6)	7.1	(6.4–7.8)	1.26	(1.18–1.34)	27,528
2011	21.0	(20.5–21.6)	10.9	(10.5–11.3)	10.1	(9.7–10.6)	16.5	(15.6–17.4)	8.6	(7.9–9.3)	7.9	(7.2–8.6)	1.28	(1.21–1.36)	29,078
2012	22.0	(21.5–22.6)	11.1	(10.7–11.5)	10.9	(10.5–11.3)	17.0	(16.2–17.9)	9.0	(8.3–9.6)	8.1	(7.4–8.7)	1.30	(1.22–1.37)	30,844
2013	23.4	(22.9–23.9)	11.5	(11.1–11.9)	11.9	(11.5–12.3)	17.6	(16.7–18.4)	9.3	(8.6–9.9)	8.3	(7.7–8.9)	1.32	(1.26–1.39)	33,210
2014	23.6	(23.1–24.2)	11.8	(11.4–12.2)	11.9	(11.4–12.3)	17.4	(16.5–18.2)	9.3	(8.6–9.9)	8.1	(7.5–8.7)	1.37	(1.30–1.44)	32,873
2015	23.2	(22.7–23.7)	11.3	(10.9–11.6)	11.9	(11.5–12.3)	16.7	(16.0–17.5)	7.8	(7.3–8.4)	8.9	(8.3–9.5)	1.38	(1.31–1.46)	35,037
2016	24.3	(23.8–24.8)	11.7	(11.3–12.0)	12.7	(12.3–13.0)	18.6	(17.8–19.3)	9.2	(8.6–9.8)	9.4	(8.8–10.0)	1.31	(1.25–1.37)	40,586
2017	25.0	(24.5–25.4)	11.6	(11.2–11.9)	13.4	(13.0–13.7)	18.9	(18.2–19.6)	8.7	(8.2–9.2)	10.2	(9.6–10.7)	1.32	(1.27–1.38)	45,456
2018	24.3	(23.9–24.8)	11.0	(10.7–11.4)	13.3	(13.0–13.7)	18.0	(17.4–18.7)	8.2	(7.7–8.7)	9.8	(9.3–10.3)	1.35	(1.30–1.41)	48,385
2019	24.9	(24.5–25.4)	11.3	(10.9–11.6)	13.7	(13.3–14.0)	18.1	(17.5–18.8)	8.3	(7.8–8.8)	9.8	(9.3–10.4)	1.37	(1.31–1.43)	51,134
2020	26.0	(25.6–26.5)	11.3	(11.0–11.6)	14.7	(14.3–15.1)	18.2	(17.6–18.9)	7.9	(7.4–8.4)	10.3	(9.8–10.8)	1.42	(1.36–1.47)	52,527
2021	26.4	(25.9–26.8)	11.9	(11.6–12.2)	14.5	(14.1–14.8)	18.6	(17.9–19.2)	8.6	(8.1–9.1)	9.9	(9.4–10.5)	1.41	(1.36–1.47)	55,797

CI: confidence interval; PR: prevalence ratio.

a Prevalence ratio of age-adjusted prevalence of overall CNS-active polypharmacy by sex estimated with incidence rate ratio from robust Poisson regression.

b Excludes subjects with missing data for sex.

c Age-adjusted prevalence of the concurrent use of ⩾ 3 CNS-active drugs with at least > 30 days of continuous exposure.

d Age-adjusted prevalence of the concurrent use of ⩾ 3 CNS-active drugs with at least > 30 days of contiguous exposure for < 180 days.

e Age-adjusted prevalence of the concurrent use of ⩾ 3 CNS-active drugs with at least > 30 days of continuous exposure for ⩾ 180 days.

### Prevalence of CNS-active polypharmacy

**Figure 1.**
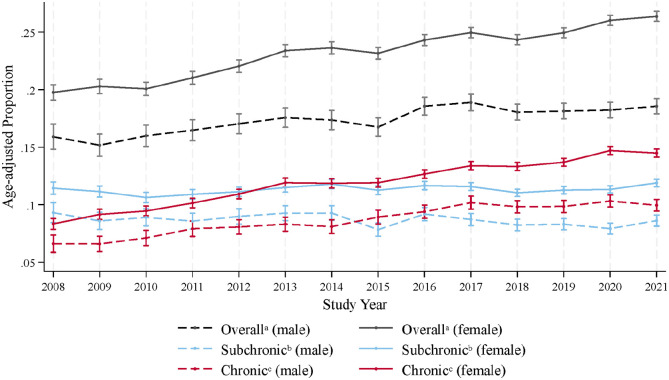
Age-adjusted prevalence of CNS-active polypharmacy among people with multiple sclerosis in the United States from 2008 to 2021 by sex. aThe concurrent use of ≥ 3 CNS-active drugs with at least > 30 days of contiguous exposure. bThe concurrent use of ≥ 3 CNS-active drugs with > 30 and <180 days of contiguous exposure. cThe concurrent use of ≥ 3 CNS-active drugs with ≥ 180 days of contiguous exposure.

**Figure 2.**
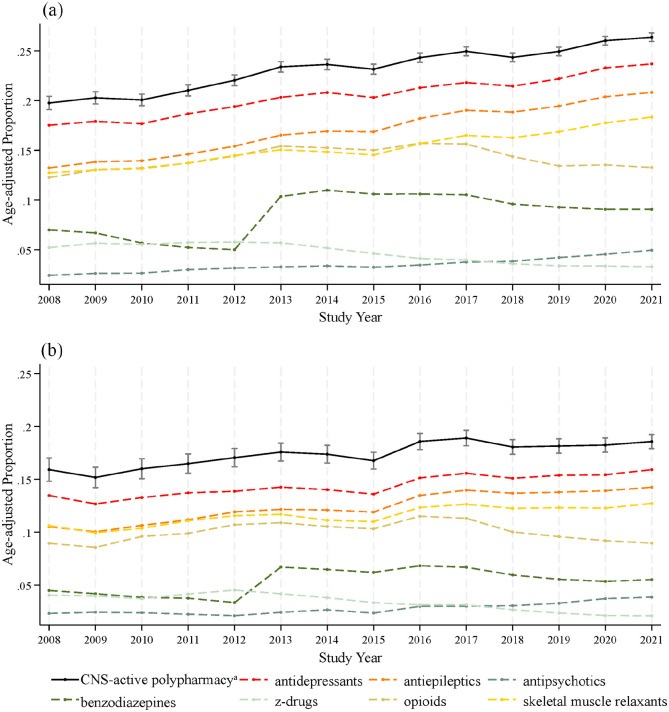
Age-adjusted prevalence of CNS-active polypharmacy among people with multiple sclerosis in the United States from 2008 to 2021 by sex with prevalence of CNS-active drug use by class (a) female and (b) male. aThe concurrent use of ≥ 3 CNS-active drugs with at least > 30 days of contiguous exposure. *Note.* 95% CI not shown for estimates of drug class prevalence.

From 2008 to 2021, the age-adjusted prevalence of CNS-active polypharmacy among female pwMS increased from 19.8% (95% CI = 19.1–20.4) to 26.4% (95% CI = 25.9–26.8) versus 15.9% (95% CI = 14.8–17.0) to 18.6% (95% CI = 17.9–19.2) in male pwMS. The age-adjusted prevalence of subchronic CNS-active polypharmacy among female pwMS changed from 11.4% (95% CI = 10.9–12.0) in 2008 to 11.9% (95% CI = 11.6–12.2) in 2021 versus 9.3% (95% CI = 8.4–10.2) to 8.6% (95% CI = 8.1–9.1) in male pwMS. Conversely, the age-adjusted prevalence of chronic CNS-active polypharmacy among female pwMS increased from 8.3% (95% CI = 7.9–8.8) in 2008 to 14.5% (95% CI = 14.1–14.8) in 2021 versus 6.6% (95% CI = 5.9–7.4) to 9.9% (95% CI = 9.4–10.5) in male pwMS. Table 1 and Figures 1 and 2 present the age-adjusted prevalence from 2008 to 2021.

### Characteristics of sample in 2021

CNS-active polypharmacy was more prevalent in male and female pwMS aged 46–55 and 56–65 years old. CNS-active polypharmacy was positively associated with rural residence in female pwMS. In male and female pwMS, CNS-active polypharmacy was associated with increased DMT utilization. Similarly, CNS-active polypharmacy was associated with increased DME utilization, the presence of an MS relapse, and MRI utilization in male and female pwMS. CNS-active polypharmacy was associated with increased comorbidities as measured by the Charlson Comorbidity Index in male and female pwMS. Similarly, CNS-active polypharmacy was associated with the presence of any CNS-related comorbidities in male (PR = 1.99 (95% CI = 1.92–2.07)) and female (PR = 1.88 (95% CI = 1.85–1.91)) pwMS (Table 2).

**Table 2.** Descriptive statistics of people with multiple sclerosis in 2021 by sex and CNS-active polypharmacy status.

	Female				PR^a^ (95% CI)	Male				PR^a^ (95% CI)
	PwMS with CNS-active polypharmacy^b^ (*n* = 10,780)		PwMS without CNS-active polypharmacy (*n* = 31,532)			PwMS with CNS-active polypharmacy^b^ (*n* = 2430)		PwMS without CNS-active polypharmacy (*n* = 11,055)		
	*n*	(%)	*n*	(%)		*n*	(%)	*n*	(%)	
Age										
18–35 years	213	(2.0)	1995	(6.3)	**0.31 (0.27–0.36)**	47	(1.9)	827	(7.5)	**0.26 (0.19–0.35)**
36–45 years	1077	(10.0)	3863	(12.3)	**0.82 (0.77–0.87)**	263	(10.8)	1468	(13.3)	**0.82 (0.72–0.92)**
46–55 years	2455	(22.8)	5849	(18.6)	**1.23 (1.18–1.28)**	565	(23.3)	2225	(20.1)	**1.16 (1.07–1.25)**
56–65 years	3702	(34.3)	7792	(24.7)	**1.39 (1.35–1.44)**	923	(38.0)	2842	(25.7)	**1.48 (1.39–1.57)**
66–75 years	2663	(24.7)	8317	(26.4)	**0.94 (0.90–0.97)**	516	(21.2)	2577	(23.3)	**0.91 (0.84–0.99)**
⩾76 years	670	(6.2)	3716	(11.8)	**0.53 (0.49–0.57)**	116	(4.8)	1116	(10.1)	**0.47 (0.39–0.57)**
US Census Region										
Midwest	2328	(21.6)	7753	(24.6)	**0.88 (0.84–0.92)**	527	(21.7)	2823	(25.5)	**0.85 (0.78–0.92)**
Northeast	1411	(13.1)	4355	(13.8)	**0.95 (0.90–1.01)**	330	(13.6)	1599	(14.5)	**0.94 (0.84–1.05)**
South	4782	(44.4)	11711	(37.1)	**1.20 (1.17–1.23)**	995	(41.0)	3924	(35.5)	**1.16 (1.10–1.22)**
West	2231	(20.7)	7632	(24.2)	**0.86 (0.82–0.90)**	574	(23.6)	2680	(24.2)	**0.97 (0.90–1.05)**
Rurality^c^										
Rural	695	(6.5)	1709	(5.4)	**1.19 (1.09–1.30)**	147	(6.1)	615	(5.6)	**1.09 (0.92–1.29)**
Nonrural	10057	(93.3)	29743	(94.3)	**0.99 (0.98–0.99)**	2279	(93.8)	10411	(94.2)	**1.00 (0.98–1.01)**
Had DMT	4590	(42.6)	11679	(37.0)	**1.20 (1.17–1.23)**	1056	(43.5)	4629	(41.9)	**1.11 (1.05–1.16)**
DMT efficacy										
High	1696	(15.7)	4220	(13.4)	**1.31 (1.24–1.38)**	469	(19.3)	1892	(17.1)	**1.27 (1.16–1.39)**
Moderate	1713	(15.9)	4090	(13.0)	**1.27 (1.21–1.34)**	356	(14.7)	1540	(13.9)	**1.11 (1.00–1.24)**
Platform	1181	(11.0)	3369	(10.7)	**1.03 (0.96–1.09)**	231	(9.5)	1197	(10.8)	**0.89 (0.78–1.02)**
Had MS relapse^d^	1222	(11.3)	1722	(5.5)	**2.09 (1.95–2.25)**	297	(12.2)	658	(6.0)	**2.08 (1.82–2.36)**
DME Use	1238	(11.5)	1915	(6.1)	**1.95 (1.82–2.08)**	398	(16.4)	767	(6.9)	**2.39 (2.13–2.67)**
Had MRI	3368	(31.2)	7891	(25.0)	**1.28 (1.24–1.32)**	695	(28.6)	2799	(25.3)	**1.17 (1.09–1.26)**
Comorbidities, CCI^e^										
None	8433	(78.2)	27623	(87.6)	**0.89 (0.88–0.90)**	1807	(74.4)	9399	(85.0)	**0.88 (0.86–0.90)**
Mild	2235	(20.7)	3746	(11.9)	**1.84 (1.75–1.92)**	570	(23.5)	1553	(14.1)	**1.73 (1.59–1.88)**
Moderate	98	(0.9)	139	(0.4)	**2.15 (1.66–2.77)**	36	(1.5)	80	(0.7)	**2.18 (1.48–3.22)**
Severe	14	(0.1)	24	(0.1)	**1.75 (0.90–3.41)**	17	(0.7)	23	(0.2)	**3.39 (1.81–6.32)**
CNS-related comorbidity										
Any CNS comorbidity	7758	(72.0)	12085	(38.3)	**1.88 (1.85–1.91)**	1695	(69.8)	3870	(35.0)	**1.99 (1.92–2.07)**
Anxiety	3088	(28.7)	3045	(9.7)	**2.97 (2.84–3.11)**	579	(23.8)	674	(6.1)	**3.93 (3.55–4.35)**
Bipolar disorder	603	(5.6)	201	(0.6)	**8.90 (7.59–10.44)**	112	(4.6)	63	(0.6)	**8.23 (6.05–11.21)**
Cognitive impairment	872	(8.1)	1104	(3.5)	**2.44 (2.24–2.66)**	250	(10.3)	427	(3.9)	**2.74 (2.36–3.18)**
Depression	4153	(38.5)	3768	(12.0)	**3.24 (3.12–3.36)**	873	(35.9)	991	(9.0)	**4.00 (3.69–4.33)**
Epilepsy/seizures	831	(7.7)	579	(1.8)	**4.21 (3.79–4.67)**	180	(7.4)	222	(2.0)	**3.68 (3.04–4.46)**
Fatigue/malaise	2596	(24.1)	4594	(14.6)	**1.66 (1.58–1.73)**	612	(25.2)	1553	(15.1)	**1.79 (1.65–1.95)**
Falls	359	(3.3)	555	(1.8)	**2.12 (1.86–2.41)**	73	(3.0)	157	(1.4)	**2.31 (1.75–3.05)**
Insomnia	1176	(10.9)	931	(3.0)	**3.71 (3.41–4.04)**	279	(11.5)	300	(2.7)	**4.23 (3.62–4.95)**
Malignancy	681	(6.3)	1730	(5.5)	**1.22 (1.12–1.33)**	179	(7.4)	725	(6.6)	**1.27 (1.09–1.48)**
Cancer pain	40	(0.4)	36	(0.1)	**3.37 (2.17–5.24)**	13	(0.5)	12	(0.1)	**5.10 (2.30–11.30)**
Noncancer pain	2590	(24.0)	2795	(8.9)	**2.71 (2.58–2.85)**	461	(19.0)	779	(7.1)	**2.72 (2.44–3.02)**
Schizophrenia	163	(1.5)	87	(0.3)	**5.48 (4.23–7.10)**	61	(2.5)	42	(0.4)	**6.83 (4.60–10.13)**
Somnolence^f^	171	(1.6)	180	(0.6)	**2.84 (2.30–3.49)**	42	(1.7)	47	(0.4)	**4.28 (2.84–6.44)**
Alcohol use disorder	161	(1.5)	164	(0.5)	**2.89 (2.32–3.59)**	76	(3.1)	118	(1.1)	**2.93 (2.20–3.90)**
Non-alcohol use disorder	588	(5.5)	275	(0.9)	**6.26 (5.43–7.22)**	175	(7.2)	150	(1.4)	**5.53 (4.46–6.86)**
Other psychotic disorders^g^	124	(1.2)	87	(0.3)	**4.28 (3.26–5.62)**	34	(1.4)	26	(0.2)	**5.95 (3.58–9.90)**

CI: confidence interval; MS: multiple sclerosis; CNS: central nervous system.

*Note*. Bold indicates statistical significance of prevalence ratio.

a The age-adjusted prevalence ratio of the respective variable by CNS-active polypharmacy status. Prevalence ratios are not age adjusted for the age variable.

b The concurrent use of ⩾ 3 CNS-active drugs with at least > 30 days of contiguous exposure.

c Rurality defined by zip code classifications from Rural-Urban Commuting Area Codes from the US Department of Agriculture. Metropolitan and micropolitan areas were categorized as nonrural while small town and rural areas were categorized as rural.

d MS relapse, or the acute worsening of neurologic function due to increased MS disease activity, was detected using MS-related inpatient, outpatient, and pharmacy claims data.

e Charlson Comorbidity Index score of 0 indicated no comorbidities, 1 to 2 indicated mild comorbidities, 3 to 4 indicated moderate comorbidities, and ⩾5 indicated severe comorbidities.

f Includes diagnosis codes for somnolence, stupor, and coma.

g Other psychotic disorders included schizotypal, delusional, brief psychotic, shared psychotic, and other or unspecified psychotic disorders.

### Characteristics associated with chronic CNS-active polypharmacy

In 2021, 56.7% (95% CI = 55.8–57.5) of subjects with CNS-active polypharmacy had chronic CNS-active polypharmacy. PwMS with chronic CNS-active polypharmacy had an average of 299.1 (95% CI = 297.7–300.4) days spent with CNS-active polypharmacy days compared to 90.0 (95% CI = 88.8–91.2) days in those with subchronic CNS-active polypharmacy. Subjects with chronic CNS-active polypharmacy had a greater number of concurrent CNS-active drugs used, unique prescribers of CNS-active drugs, and unique prescriber types (Table 3).

**Table 3.** Person-days, CNS-active polypharmacy days, number of concurrent CNS-active-drugs prescribed, and number of prescribers and prescriber types prescribing drugs contributing to CNS-active polypharmacy in people with multiple sclerosis in 2021.

	PwMS with chronic CNS-active polypharmacy^a^ (*n* = 7488)			PwMS with subchronic CNS-active polypharmacy^b^ (*n* = 5722)		
	M	(SD)	(95% CI)	M	(SD)	(95% CI)
Person-days^c^	353.9	(0.4)	(353.2–354.6)	296.4	(1.3)	(293.8.-299.0)
Polypharmacy days^d^	299.1	(0.7)	(297.7–300.4)	90.0	(0.6)	(88.8–91.2)
	*n*	(%)	(95% CI)	*n*	(%)	(95% CI)
No. CNS-active drugs						
3	1110	(14.8)	(14.0–15.7)	2731	(47.7)	(46.4–49.0)
4	2214	(29.6)	(28.5–30.6)	1813	(31.7)	(30.5–32.9)
5	1955	(26.1)	(25.1–27.1)	782	(13.7)	(12.8–14.6)
6	1184	(15.8)	(15.0–16.7)	253	(4.4)	(3.9–5.0)
7	593	(7.9)	(7.3–8.6)	94	(1.6)	(1.3–2.0)
⩾8	432	(5.8)	(5.3–6.3)	49	(0.9)	(0.7–1.1)
No. prescribers^e^						
1	965	(12.9)	(12.2–13.7)	1344	(23.5)	(22.4–24.6)
2	1675	(22.4)	(21.4–23.3)	2033	(35.5)	(34.3–36.8)
3	1661	(22.2)	(21.3–23.1)	1327	(23.2)	(22.1–24.3)
4	1231	(16.4)	(15.6–17.3)	606	(10.6)	(9.8–11.4)
5	844	(11.3)	(10.6–12.0)	259	(4.5)	(4.0–5.1)
6	495	(6.6)	(6.1–7.2)	91	(1.6)	(1.3–2.0)
7	283	(3.8)	(3.4–4.2)	37	(0.7)	(0.5–0.9)
⩾ 8	334	(4.5)	(4.0–5.0)	25	(0.4)	(0.3–0.7)
No. prescriber types^f^						
1	1163	(15.5)	(14.7–16.4)	1536	(26.8)	(25.7–28.0)
2	1982	(26.5)	(25.5–27.5)	2153	(37.6)	(36.4–38.9)
3	1828	(24.4)	(23.5–25.4)	1274	(22.3)	(21.2–23.4)
4	1211	(16.2)	(15.4–17.0)	497	(8.7)	(8.0–9.4)
5	685	(9.2)	(8.5–9.8)	189	(3.3)	(2.9–3.8)
6	333	(4.5)	(4.0–4.9)	55	(1.0)	(0.7–1.3)
7	152	(2.0)	(1.7–2.4)	13	(0.2)	(0.1–0.4)
⩾8	134	(1.8)	(1.5–2.1)	5	(0.1)	(0.0–0.2)

SD: standard deviation; CI: confidence interval.

a The concurrent use of ⩾ 3 CNS-active drugs with at least > 30 days of contiguous exposure.

b The concurrent use of ⩾ 3 CNS-active drugs with at least > 30 days of contiguous exposure for ⩾ 180 days total.

c Total days where a subject had insurance coverage for a period of > 30 days in 2021.

d Total days where a subject had CNS-active polypharmacy for > 30 days in 2021.

e Number of unique providers that prescribed CNS-active drugs that contributed to CNS-active polypharmacy.

f Number of unique type of providers that prescribed CNS-active drugs contributing to polypharmacy (e.g. neurologists, internists, nurse practitioners).

Compared to subchronic CNS-active polypharmacy, chronic CNS-active polypharmacy was more prevalent in female pwMS 46–55 years old and male pwMS 56–65 years old (Supplemental eTable 4) and was not associated with rural residence. DMT utilization, presence of a relapse, DME utilization, and MRI utilization were positively associated with chronic CNS-active polypharmacy only in female pwMS. In male and female pwMS, mild comorbidities and the presence of any CNS-related comorbidity were positively associated with chronic CNS-active polypharmacy. Prevalence ratios for each of the descriptive variables by chronicity are presented in Supplemental eTable 4.

### CNS-active drugs commonly prescribed

Gabapentin and baclofen were the two most prescribed CNS-active drugs that contributed to chronic and subchronic CNS-active polypharmacy. The age and sex-adjusted prevalence ratio between chronic and subchronic CNS-active polypharmacy for gabapentin was 1.19 (95% CI = 1.15-1.23) and 1.24 (95% CI = 1.19-1.29) for baclofen. Sertraline and escitalopram had no statistical evidence of increased prevalence in pwMS with chronic CNS-active polypharmacy compared to those with subchronic CNS-active polypharmacy (Supplemental eTable 5). Oxycodone and hydrocodone were among the most likely drugs to be present in subjects with chronic versus subchronic CNS-active polypharmacy (Supplemental eTable 5).

### Prescribers contributing to CNS-active polypharmacy

CNS-active drug prescriptions contributing to CNS-active polypharmacy resulted in 4,772,033 cumulative days of drug supply for pwMS in 2021. Prescriptions from primary care physicians and neurologists, respectively, comprised 38.2% and 24.2% of the days of CNS-active drug supply. Primary care physicians were the most frequent prescriber of antidepressants, antipsychotics, benzodiazepines, z-drugs, and opioids. Neurologists were the most frequent prescriber of antiepileptics and skeletal muscle relaxants (Supplemental eTable 6).

## Discussion

In this serial cross-sectional analysis of large commercial and Medicare Advantage health plans, CNS-active polypharmacy was increasingly prevalent among pwMS. By 2021, the age-adjusted prevalence of CNS-active polypharmacy among female pwMS increased to 26.4% versus to 18.6% in male pwMS. However, the observed increase in overall CNS-active polypharmacy was confounded by the chronicity of exposure as chronic exposure (⩾ 180 days) increased over time while subchronic exposure (< 180 days) remained stable. Female pwMS consistently had a greater prevalence of CNS-active polypharmacy, perhaps explained by an increased diagnosis and treatment of CNS-related comorbidities compared to male pwMS.

Comparison of our findings is limited by the scarcity of CNS-active polypharmacy studies in pwMS. One study of Norwegian pwMS using ⩾ 1 antiepileptic drug found that 60% used ⩾ 1 additional CNS-active drug at a MS Rehabilitation Center from 2009 to 2012.^20^ While this study found a high prevalence of concurrent CNS-active drug use in a rehabilitation setting, our study detected a high and increasing prevalence of the chronic use of > 3 CNS-active drugs of any class across inpatient and outpatient settings.

The increasing prevalence of CNS-active polypharmacy has been detected in other populations. Among older adults in the US represented in the National Ambulatory Medical Care Survey, the prevalence increased from 0.6% to 1.4% from 2004 to 2013.^12,13^ However, adult National Health and Nutrition Examination Survey (NHANES) participants showed no increase in the prevalence of CNS-active polypharmacy from 2009 to 2020.^10^ Our study enhances these findings, suggesting that prescription patterns for chronic CNS-related disease may explain the increasing burden of CNS-active polypharmacy. For example, the persistent increase in chronic CNS-active polypharmacy in pwMS may more likely represent an increase in the clinical priority to treat depression and anxiety in a population already using skeletal muscle relaxants and antiepileptics to manage pain and spasticity. Conversely, the stable prevalence of subchronic CNS-active polypharmacy may be explained by appropriate, event-related prescriptions—such as opioid analgesics following surgery in pwMS already treated for depression and spasticity.

In our study, the female-to-male age-adjusted prevalence ratio of CNS-active polypharmacy increased from 1.27 to 1.41 indicating an increasing disparity by sex. Previous estimates of CNS-active polypharmacy in the general population found an increased burden among females (3.3%) versus males (2.0%); however, the magnitude of the disparity did not change significantly from 2009 to 2020.^10^ The increased diagnosis of specific CNS-related comorbidities in pwMS may partially explain this difference. For example, pain is a common symptom of MS, regularly presenting as continuous neuropathic pain and painful spasticity. Among female pwMS CNS-active polypharmacy, the prevalence of pain was 24.0%—approximately 2.7 times as prevalent compared to those without polypharmacy. While pain was similarly 2.7 times as prevalent in male pwMS with versus without CNS-active polypharmacy, the absolute prevalence of pain was lower (19.0%). While the age-adjusted likelihood of having any CNS-active comorbidity was higher in male pwMS with CNS-active polypharmacy (PR = 1.99) compared to female pwMS with CNS-active polypharmacy (PR = 1.88), the absolute prevalence of any CNS-related comorbidity remained higher in female pwMS (72.0%), likely explaining the role of comorbidity in CNS-active polypharmacy.

CNS-active comorbidities may modulate the severity of MS and increase CNS-active drug use. For example, depression was highly prevalent in male and female pwMS in our study and has been associated with significantly higher sustained Expanded Disability Status Scale (EDSS) scores in pwMS.^26^ Our study accordingly detected a higher prevalence of potential indicators of disease severity or activity, such as DMT utilization, presence of algorithm-derived relapses, MRI utilization, and DME utilization. Thus, the association between MS severity and depression could partially explain the observed high prevalence of antidepressants in our study.

Gabapentin and baclofen, indicated for MS-related neuropathic pain and spasticity, respectively, were highly prevalent in both subchronic and chronic CNS-active polypharmacy. Our results also highlight the contribution of opioid use to chronic CNS-active polypharmacy. Here, hydrocodone and oxycodone were, respectively, 1.60 and 1.69 times more likely to contribute to chronic CNS-active polypharmacy than subchronic. Opioid prescription may be inappropriate in pwMS when used in lieu of medications targeting neuropathic pain or spasticity or non-pharmacologic approaches to chronic pain. Chronic opioid use is associated with increased constipation and sleep-disordered breathing, conditions commonly experienced by pwMS, plausibly contributing to increased morbidity in this population.^27–31^

In 2021, pwMS with chronic CNS-active polypharmacy had the greatest number of prescribers. Primary care physicians were the most common prescriber of CNS-active drugs contributing to CNS-active polypharmacy followed by neurologists. However, primary care physicians highly contributed to prescriptions in all drug classes while neurologists selectively prescribed antiepileptics and skeletal muscle relaxants indicated for the treatment of symptoms of MS. The complexity of the coordination of appropriate care across multiple prescribers may result in cases of potentially inappropriate CNS-active polypharmacy.

### Limitations

This study was limited by its convenience sample from a single commercial insurer as the CDM has demonstrated selection against people who are younger, Black, Hispanic, or of lower socioeconomic status.^32^ The prevalence of CNS-active polypharmacy is likely overestimated as insured pwMS likely have a greater chance to receive prescriptions compared to uninsured pwMS. Income, education, race, and ethnicity data were not available in this study, so variations in the prevalence of CNS-active polypharmacy by these factors were unable to be investigated. While the algorithm used to identify pwMS has a high demonstrated positive predictive value (99%) negative predictive value (96%) in a high prevalence population, pwMS with less severe disease may be excluded.^22^ Similarly, algorithms used to estimate the prevalence of DMT use, CNS-related comorbidities, and MS relapses may result in misclassification. The use of prescription data to measure CNS-active drug use likely overestimates CNS-active drug use when prescriptions were filled but not taken daily. Moreover, drug utilization not represented in the insurance database was unable to be measured nor was the appropriateness of each prescription. Finally, this study was unable to measure the outcomes of CNS-active polypharmacy due to the cross-sectional design. Future studies may overcome these limitations using multiple data sources in a cohort design.

## Conclusion

In this serial cross-sectional analysis from 2008 to 2021, the age-adjusted prevalence of CNS-active polypharmacy was highly prevalent among female pwMS increasing from 19.8% to 26.4% versus 15.9% to 18.6% in male pwMS. The female-to-male age-adjusted prevalence ratio increased from 1.27 to 1.41 indicating an increasing disparity by sex. The coordination of complex care for pwMS must be carefully planned to minimize the harms associated with CNS-active polypharmacy.

## Supplemental Material

sj-csv-1-msj-10.1177_13524585241251986 – Supplemental material for Trends in central nervous system-active polypharmacy among people with multiple sclerosis Supplemental material, sj-csv-1-msj-10.1177_13524585241251986 for Trends in central nervous system-active polypharmacy among people with multiple sclerosis by Hayden Naizer, Joseph Wozny, Trudy Millard Krause, Ethan Huson and Leorah Freeman in Multiple Sclerosis Journal

sj-docx-2-msj-10.1177_13524585241251986 – Supplemental material for Trends in central nervous system-active polypharmacy among people with multiple sclerosis Supplemental material, sj-docx-2-msj-10.1177_13524585241251986 for Trends in central nervous system-active polypharmacy among people with multiple sclerosis by Hayden Naizer, Joseph Wozny, Trudy Millard Krause, Ethan Huson and Leorah Freeman in Multiple Sclerosis Journal

sj-docx-3-msj-10.1177_13524585241251986 – Supplemental material for Trends in central nervous system-active polypharmacy among people with multiple sclerosis Supplemental material, sj-docx-3-msj-10.1177_13524585241251986 for Trends in central nervous system-active polypharmacy among people with multiple sclerosis by Hayden Naizer, Joseph Wozny, Trudy Millard Krause, Ethan Huson and Leorah Freeman in Multiple Sclerosis Journal

sj-xlsx-4-msj-10.1177_13524585241251986 – Supplemental material for Trends in central nervous system-active polypharmacy among people with multiple sclerosis Supplemental material, sj-xlsx-4-msj-10.1177_13524585241251986 for Trends in central nervous system-active polypharmacy among people with multiple sclerosis by Hayden Naizer, Joseph Wozny, Trudy Millard Krause, Ethan Huson and Leorah Freeman in Multiple Sclerosis Journal

## References

[1] Murphy K Bethea JR Fischer R . Neuropathic pain in multiple sclerosis—Current therapeutic intervention and future treatment perspectives. In: Zagon IS McLaughlin PJ (eds) Multiple sclerosis: Perspectives in treatment and pathogenesis. Brisbane, QLD, Australia: Codon Publications, 2017, pp. 53–69. 29261265

[2] Marrie RA Fisk JD Walld R , et al. Prescription opioid use in multiple sclerosis. J Neurol Neurosurg Psychiatry 2022; 94(2): 167–169. 36028309 10.1136/jnnp-2022-329508 PMC9872229

[3] Ehde DM Alschuler KN Osborne TL , et al. Utilization and patients’ perceptions of the effectiveness of pain treatments in multiple sclerosis: A cross-sectional survey. Disabil Health J 2015; 8(3): 452–456. 25899795 10.1016/j.dhjo.2015.03.001 PMC4464976

[4] Otero-Romero S Sastre-Garriga J Comi G , et al. Pharmacological management of spasticity in multiple sclerosis: Systematic review and consensus paper. Mult Scler 2016; 22(11): 1386–1396. 27207462 10.1177/1352458516643600

[5] Marrie RA Cohen J Stuve O , et al. A systematic review of the incidence and prevalence of comorbidity in multiple sclerosis: Overview. Mult Scler J 2015; 21(3): 263–281. 10.1177/1352458514564491 PMC4361468 25623244

[6] Foley PL Vesterinen HM Laird BJ , et al. Prevalence and natural history of pain in adults with multiple sclerosis: Systematic review and meta-analysis. Pain 2013; 154(5): 632–642. 23318126 10.1016/j.pain.2012.12.002

[7] 2023 American Geriatrics Society Beers Criteria^®^ Update Expert Panel. American Geriatrics Society 2023 updated AGS Beers Criteria^®^ for potentially inappropriate medication use in older adults. J Am Geriatr Soc 2023; 71: 2052–2081. 37139824 10.1111/jgs.18372 PMC12478568

[8] Fick DM Semla TP Steinman M , et al. American Geriatrics Society 2019 updated AGS Beers Criteria^®^ for potentially inappropriate medication use in older adults. J Am Geriatr Soc 2019; 67(4): 674–694. 30693946 10.1111/jgs.15767

[9] American Geriatrics Society 2015 Beers Criteria Update Expert Panel. American Geriatrics Society 2015 updated beers criteria for potentially inappropriate medication use in older adults. J Am Geriatr Soc 2015; 63(11): 2227–2246. 26446832 10.1111/jgs.13702

[10] Terman SW Niznik JD Growdon ME , et al. Secular trends in central nervous system-active polypharmacy among serial cross-sections of US adults, 2009–2020. Drugs Aging 2023; 40(10): 941–951. 37695395 10.1007/s40266-023-01066-w PMC10629698

[11] Terman SW Aubert CE Hill CE , et al. Polypharmacy in patients with epilepsy: A nationally representative cross-sectional study. Epilepsy Behav 2020; 111: 107261. 32629416 10.1016/j.yebeh.2020.107261 PMC7869064

[12] Gerlach LB Olfson M Kales HC , et al. Opioids and other central nervous system-active polypharmacy in older adults in the United States. J Am Geriatr Soc 2017; 65(9): 2052–2056. 28467623 10.1111/jgs.14930 PMC5603361

[13] Maust DT Gerlach LB Gibson A , et al. Trends in central nervous system-active polypharmacy among older adults seen in outpatient care in the United States. JAMA Intern Med 2017; 177(4): 583–585. 28192559 10.1001/jamainternmed.2016.9225 PMC5378654

[14] Hart LA Phelan EA Yi JY , et al. Use of fall risk–Increasing drugs around a fall-related injury in older adults: A systematic review. J Am Geriatr Soc 2020; 68(6): 1334–1343. 32064594 10.1111/jgs.16369 PMC7299782

[15] Park TW Saitz R Ganoczy D , et al. Benzodiazepine prescribing patterns and deaths from drug overdose among US veterans receiving opioid analgesics: Case-cohort study. BMJ 2015; 350: h2698. 10.1136/bmj.h2698 PMC4462713 26063215

[16] Dunn KM Saunders KW Rutter CM , et al. Overdose and prescribed opioids: Associations among chronic non-cancer pain patients. Ann Intern Med 2010; 152: 85–92.20083827

[17] Hart LA Walker R Phelan EA , et al. Change in central nervous system-active medication use following fall-related injury in older adults. J Am Geriatr Soc 2022; 70(1): 168–177. 34668191 10.1111/jgs.17508 PMC8742750

[18] Porsteinsson AP Drye LT Pollock BG , et al. Effect of citalopram on agitation in Alzheimer disease: The CitAD randomized clinical trial. JAMA 2014; 311(7): 682–691. 24549548 10.1001/jama.2014.93 PMC4086818

[19] Cortese M Bjornevik K Chitnis T , et al. Aging with multiple sclerosis: A longitudinal study of physical function, mental health, and memory in two cohorts of US women. Mult Scler 2022; 28(1): 121–131. 33860717 10.1177/13524585211007739 PMC8521558

[20] Beiske GAG Holmøy T Beiske AG , et al. Antiepileptic and antidepressive polypharmacy in patients with multiple sclerosis. Mult Scler Int 2015; 2015: 317859. 26221541 10.1155/2015/317859 PMC4499608

[21] Clinformatics^®^ Data Mart Database. Optum; 2007-2021. Accessed June 15, 2023. https://www.optum.com/

[22] Culpepper WJ Marrie RA Langer-Gould A , et al. Validation of an algorithm for identifying MS cases in administrative health claims datasets. Neurology 2019; 92(10): e1016–e1028. 10.1212/WNL.0000000000007043 PMC6442008 30770432

[23] Maust DT Strominger J Kim HM , et al. Prevalence of central nervous system-active polypharmacy among older adults with dementia in the US. JAMA 2021; 325(10): 952–961. 33687462 10.1001/jama.2021.1195 PMC7944381

[24] Freeman L Longbrake EE Coyle PK , et al. High-efficacy therapies for treatment-naïve individuals with relapsing-remitting multiple sclerosis. CNS Drugs 2022; 36(12): 1285–1299. 36350491 10.1007/s40263-022-00965-7 PMC9645316

[25] Chastek BJ Oleen-Burkey M Lopez-Bresnahan MV . Medical chart validation of an algorithm for identifying multiple sclerosis relapse in healthcare claims. J Med Econ 2010; 13(4): 618–625. 20883151 10.3111/13696998.2010.523670

[26] Binzer S McKay KA Brenner P , et al. Disability worsening among persons with multiple sclerosis and depression: A Swedish cohort study. Neurology 2019; 93(24): e2216–e2223. 10.1212/WNL.0000000000008617 PMC6937491 31704791

[27] Walker JM Farney RJ Rhondeau SM , et al. Chronic opioid use is a risk factor for the development of central sleep apnea and ataxic breathing. J Clin Sleep Med 2007; 3(5): 455–461. 17803007 PMC1978331

[28] Farney RJ McDonald AM Boyle KM , et al. Sleep disordered breathing in patients receiving therapy with buprenorphine/naloxone. Eur Respir J 2013; 42(2): 394–403. 23100497 10.1183/09031936.00120012

[29] FitzHenry F Eden SK Denton J , et al. Prevalence and risk factors for opioid-induced constipation in an older national veteran cohort. Pain Res Manag 2020; 2020: 5165682. 32318129 10.1155/2020/5165682 PMC7149448

[30] Preziosi G Gordon-Dixon A Emmanuel A . Neurogenic bowel dysfunction in patients with multiple sclerosis: Prevalence, impact, and management strategies. Degener Neurol Neuromuscul Dis 2018; 8: 79–90. 30584387 10.2147/DNND.S138835 PMC6287516

[31] Braley TJ Segal BM Chervin RD . Sleep-disordered breathing in multiple sclerosis. Neurology 2012; 79: 929–936. 22895593 10.1212/WNL.0b013e318266fa9d PMC3425840

[32] Dahlen A Charu V . Analysis of sampling bias in large health care claims databases. JAMA Netw Open 2023; 6(1): e2249804. 10.1001/jamanetworkopen.2022.49804 PMC9857613 36607640

