# Supplementary material for: Trends in central nervous system-active polypharmacy among people with multiple sclerosis
Source: Mult Scler. 2024 May 15;30(9):1139–50. doi: 10.1177/13524585241251986 (PMC11363472; doi:10.1177/13524585241251986)
Supplement: sj-docx-2-msj-10.1177_13524585241251986 – Supplemental material for Trends in central nervous system-active polypharmacy among people with multiple sclerosis [file sj-docx-2-msj-10.1177_13524585241251986.docx]

Supplementary Materials

# eTable1: Disease modifying therapies included in the identification of people with MS or measurement of DMT utilization

| **DMT** | **Efficacy** | **NDC** | **HCPC** |
| --- | --- | --- | --- |
| Alemtuzumab | High | 50419035510, 50419035512, 50419035703, 50419355110, 50419355112, 58468020001, 58468035701, 58468035703 | J0202 |
| Cladribine | High | 14398710100, 42658001001, 44087400000, 44087400004, 44087400005, 44087400006, 44087400007, 44087400008, 44087400009, 55390011501, 55390012401, 59676020101, 63323014010, 67457045010, 67457045110, 69008618000, 69020101000 | J9065 |
| Dimethyl fumarate | Moderate | 16729041604, 16729041712, 16729041759, 24979012721, 24979012804, 31722065731, 31722065832, 31722068060, 37803961400, 37803991800, 37803999100, 43598042952, 43598043060, 51407044160, 51407044214, 64406000501, 64406000602, 64406000703, 67877055514, 67877055532, 67877055623, 67877055660, 67877055739, 68180077614, 68180077665, 68180077707, 68180077748, 68180077813, 69097032228, 69097032289, 69097032303, 69097032388, 69097055203, 69238131804, 69238131906, 69238162603, 93921841000, 93921906000 |  |
| Diroximel fumarate | Moderate | 64406002001, 64406002003, 65757002003 |  |
| Fingolimod | Moderate | 16729034210, 31722088930, 37845259300, 43598028530, 60505433203, 62756006483, 64980044903, 67877047630, 68382091206, 68462016630, 70709006230, 70709006530, 78060715000, 78060751000, 78060789000, 78096589000 |  |
| Glatiramer acetate | Platform | 37869603200, 37869609300, 37869611200, 37869613200, 63629881501, 63629881601, 68115075030, 68546031730, 68546032506, 68546032512, 78132343400, 78132347100, 78132507100, 78132508900, 88115003000, 88115330000 | J1595 |
| Interferon beta-1a, Pegylated interferon beta-1a | Platform | 44087002203, 44087002209, 44087004403, 44087004409, 44087018801, 44087332201, 44087332209, 44087334401, 44087334409, 44087882201, 54569443300, 59627000205, 59627000206, 59627000207, 59627000301, 59627000304, 59627011103, 59627022205, 59627033304, 64406001101, 64406001201, 64406001501, 64406001601, 64406001701 | J1826,  Q3028 |
| Interferon beta-1b | Platform | 50419052103, 50419052115, 50419052201, 50419052309, 50419052315, 50419052325, 50419052335, 50419052401, 50419052435, 78056912000, 78056961000, 78056999000 | J1830 |
| Monomethyl fumarate | Moderate | 69387000101 |  |
| Natalizumab^a^ | High | 59075073015, 64406000801 | J2323 |
| Ocrelizumab | High | 50242015001 | J2350 |
| Ofatumumab | High | 17308080200, 17308080500, 17308210100, 17308210200, 17308213300, 78066913000, 78066961000, 78069061000, 78100768000 | J9302 |
| Ozanimod | Moderate | 59572081007, 59572082030, 59572089007, 59572089030, 59572089091 |  |
| Ponisemod | Moderate | 50458070714, 50458072030 |  |
| Rituximab^b^ | High | 50242005110, 50242005121, 50242005306, 50242010801, 50242010901, 55513022401, 55513032601, 63459010310, 63459010450, 69023801000, 69024901000 | J9311, J9312, Q5115, Q5119, Q5123 |
| Siponimod | Moderate | 78097912000, 78097950000, 78097989000, 78098615000, 78098645000, 78101415000 |  |
| Teriflunomide | Moderate | 58468021001, 58468021002, 58468021004, 58468021101, 58468021102, 58468021104 |  |
| Footnote: a - Natalizumab claims were excluded from identification if a person had any claim with a diagnosis code indicating irritable bowel disease. b - Rituximab utilization was excluded from patient identification. | | | |

eTable 2: CNS-active drugs included using the American Hospital Formulary Service (AHFS) Pharmacologic-Therapeutic Classification System

| **CNS-active drug class** | **AHFS code** | **Drug Name** |
| --- | --- | --- |
| Antidepressants | 28160420 | Amitriptyline, Amoxapine, Bupropion, Citalopram, Clomipramine, Desipramine, Desvenlafaxine, Doxepin, Duloxetine, Escitalopram, Esketamine, Fluoxetine, Fluvoxamine, Imipramine, Isocarboxazid, Levomilnacipran, Maprotiline, Mirtazapine, Nefazodone, Nortriptyline, Paroxetine, Phenelzine, Protriptyline, Sertraline, Tranylcypromine, Trazodone, Trimipramine, Venlafaxine, Vilazodone, Vortioxetine |
|  | 28160416 |  |
|  | 28160428 |  |
|  | 28160412 |  |
|  | 28160492 |  |
|  | 28160424 |  |
| Antiepileptics | 28120400 | Brivaracetam, Cannabidiol, Carbamazepine, Cenobamate, Divalproex, Eslicarbazepine, Ethosuximide, Ezogabine, Felbamate, Gabapentin, Lacosamide, Lamotrigine, Levetiracetam, Magnesium, Mephobarbital, Oxcarbazepine, Perampanel, Phenytoin, Pregabalin, Primidone, Tiagabine, Topiramate, Valproate, Zonisamide |
|  | 28121200 |  |
|  | 28129200 |  |
| Antipsychotics | 28160804 | Aripiprazole, Asenapine, Brexpiprazole, Cariprazine, Chlorpromazine, Clozapine, Fluphenazine, Haloperidol, Iloperidone, Loxapine, Lumateperone, Lurasidone, Olanzapine, Paliperidone, Perphenazine, Pimavanserin, Pimozide, Quetiapine, Risperidone, Thioridazine, Thiothixene, Trifluoperazine, Ziprasidone |
|  | 28160808 |  |
|  | 28160892 |  |
|  | 28160824 |  |
|  | 28160832 |  |
| Benzodiazepines | 28240800 | Alprazolam, Chlordiazepoxide, Clorazepate, Diazepam, Estazolam, Flurazepam, Lorazepam, Midazolam, Oxazepam, Quazepam, Temazepam, Triazolam |
|  |  |  |
| Nonbenzodiazepine, benzodiazepine receptor agonist hypnotics (“z-drugs”) | 28249200 | Eszopiclone, Zaleplon, Zolpidem |
|  |  |  |
| Opioids | 28080800 | Buprenorphine, Butorphanol, Codeine, Fentanyl, Hydrocodone, Levorphanol, Meperidine, Methadone, Morphine, Nalbuphine, Opium, Oxycodone, Oxymorphone, Pentazocine, Propoxyphene, Tapentadol, Tramadol |
|  | 28081200 |  |
| Skeletal muscle relaxants | 12201200 | Baclofen, Carisoprodol, Chlorzoxazone, Cyclobenzaprine, Dantrolene, Metaxalone, Methocarbamol, Orphenadrine, Tizanidine |
|  | 12200400 |  |
|  | 12200800 |  |
|  | 12209200 |  |
|  | | |

eTable 3: CNS related comorbidities included using ICD-10-CM codes^1^

| **Disorder** | **ICD-10-CM codes** |
| --- | --- |
| Anxiety | 'F064', 'F40', 'F400', 'F4001', 'F4002', 'F401', 'F4010', 'F4011', 'F402', 'F4021', 'F4022', 'F2023', 'F4024', 'F4029', 'F408', 'F409', 'F41', 'F410', 'F413', 'F418', 'F419', 'F42', 'F43', 'F430', 'F438', 'F439', 'F452', 'F4520', 'F4521', 'F4529', 'F457', 'F431', 'F4310', 'F4311', 'F4312' |
| Bipolar disorder | 'F301', 'F302', 'F303', 'F304', 'F308', 'F309', 'F310', 'F311', 'F312', 'F313', 'F314', 'F315', 'F316', 'F317', 'F318', 'F319', 'F340' |
| Cognitive impairment | 'R418', 'G3184' |
| Depression | 'F32', 'F320', 'F321', 'F322', 'F323', 'F324', 'F325', 'F329', 'F33', 'F330', 'F331', 'F332', 'F333', 'F334', 'F3340', 'F3341', 'F3342', 'F338', 'F339' |
| Epilepsy/seizures | 'G40', 'G41' |
| Fatigue/malaise | 'R53' |
| Falls | 'W0', 'W1' |
| Insomnia | 'G4700', 'G4701', 'G4709' |
| Malignancy | 'C00' – 'C96' |
| Cancer pain | 'G893' |
| Noncancer pain | See supplemental dataset “Noncancer pain ICD-10-CM” |
| Schizophrenia | 'F200', 'F201', 'F202', 'F203', 'F205', 'F208', 'F209', 'F250', 'F251', 'F258', 'F259' |
| Somnolence | 'R40' |
| Alcohol use disorder | 'F101','F102','F109' |
| Non-alcohol use disorder | 'F111', 'F112', 'F141', 'F142', 'F149', 'F151', 'F152', 'F159', 'F131', 'F132', 'F139', 'F121', 'F122', 'F129', 'F161', 'F162', 'F169', 'F181', 'F182', 'F189', 'F191', 'F192', 'F199' |
| Other psychotic disorders | 'F21','F22','F23','F24','F28','F29' |
| 1. ICD-10-CM codes derived from: Maust DT, Strominger J, Kim HM, et al. Prevalence of Central Nervous System-Active Polypharmacy Among Older Adults With Dementia in the US. *JAMA*. 2021;325(10):952-961. doi:10.1001/jama.2021.1195 | |

eFigure 1: Flow diagram of subject selection in 2021


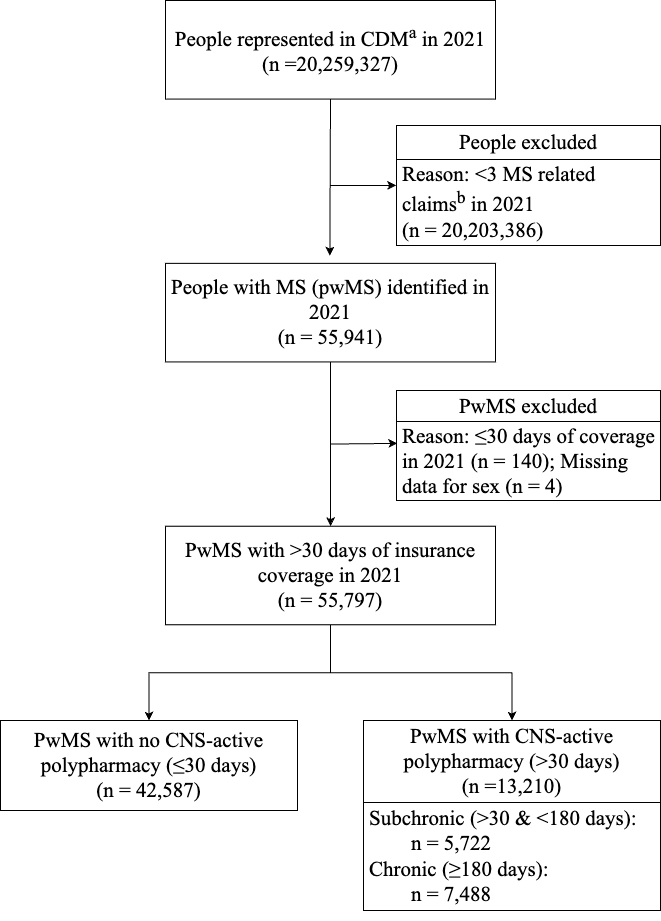


Footnote: a –Optum’s de-identified Clinformatics^®^ Data Mart (CDM) is derived from a database of administrative health claims for members of large commercial and Medicare Advantage health plans b – A validated algorithm for identifying MS patients in claims data: (Inpatient Episodes + Outpatient Claims + Disease Modifying Therapy Claims (DMT)) ≥ 3 within one year.

# eFigure 2: Age-adjusted prevalence of CNS-active polypharmacy among people with multiple sclerosis in the United States from 2008 to 2021 by sex and chronicity of exposure with prevalence of CNS-active drug use by class

|  | Female | Male |
| --- | --- | --- |
| Chronic | 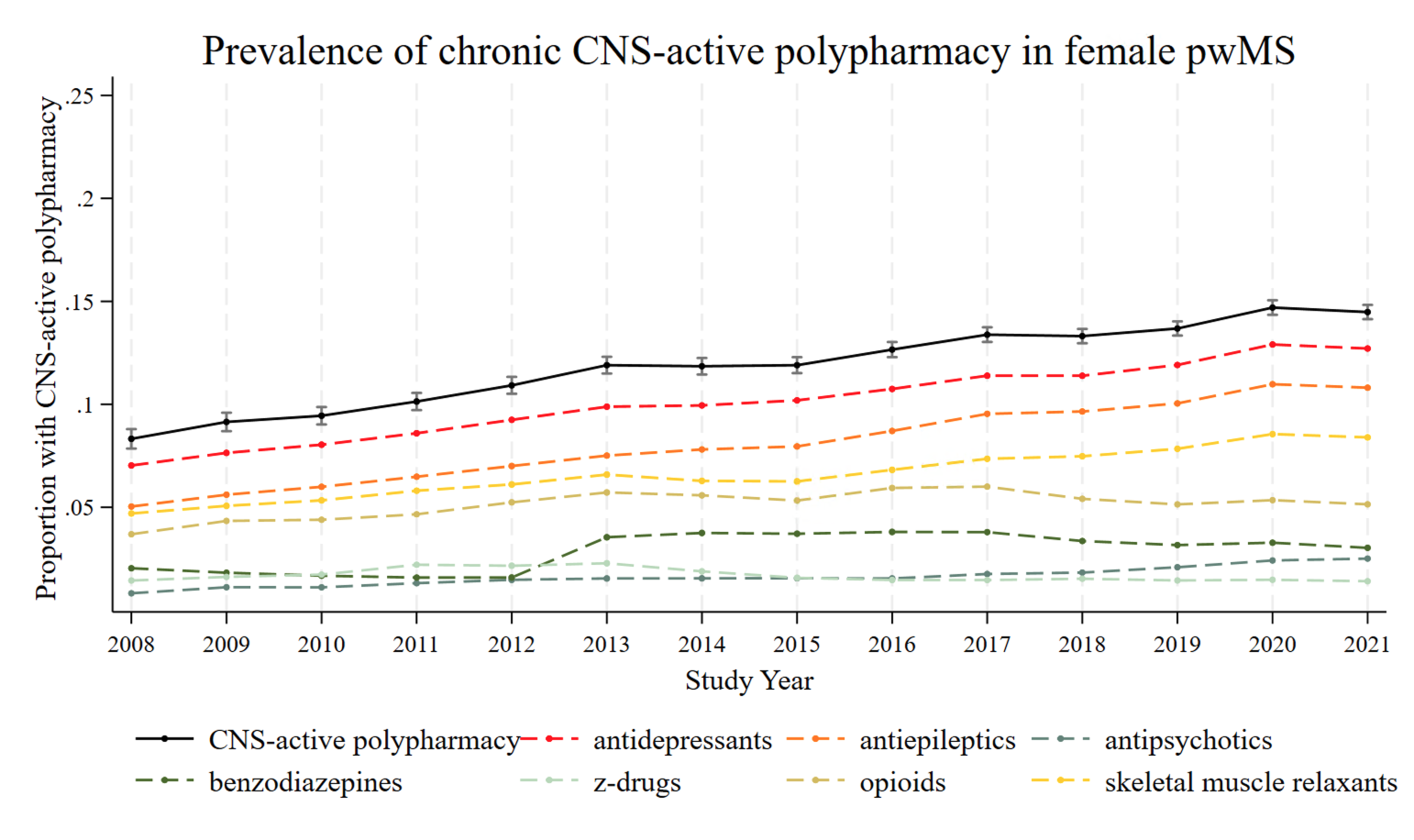 | 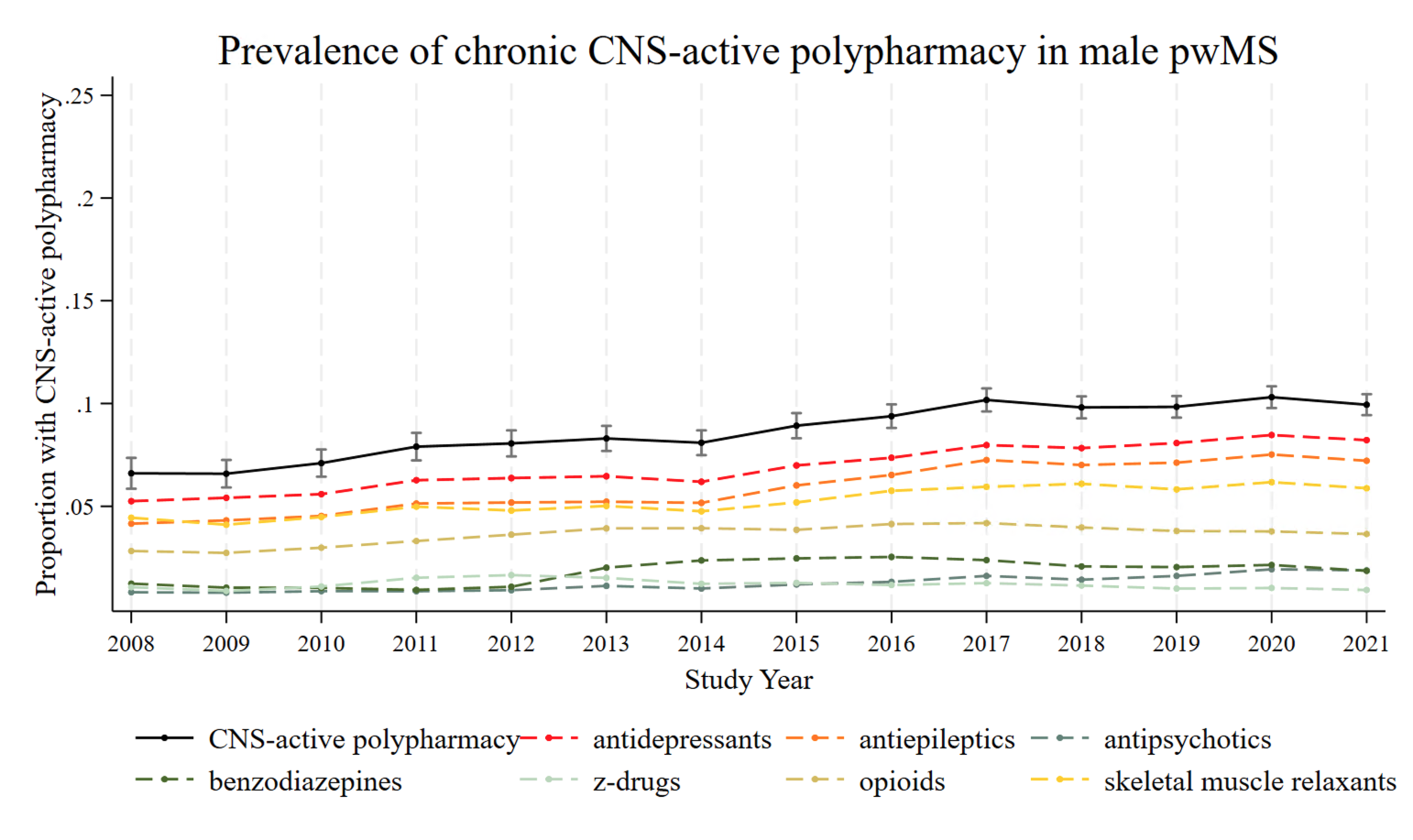 |
| Subchronic | 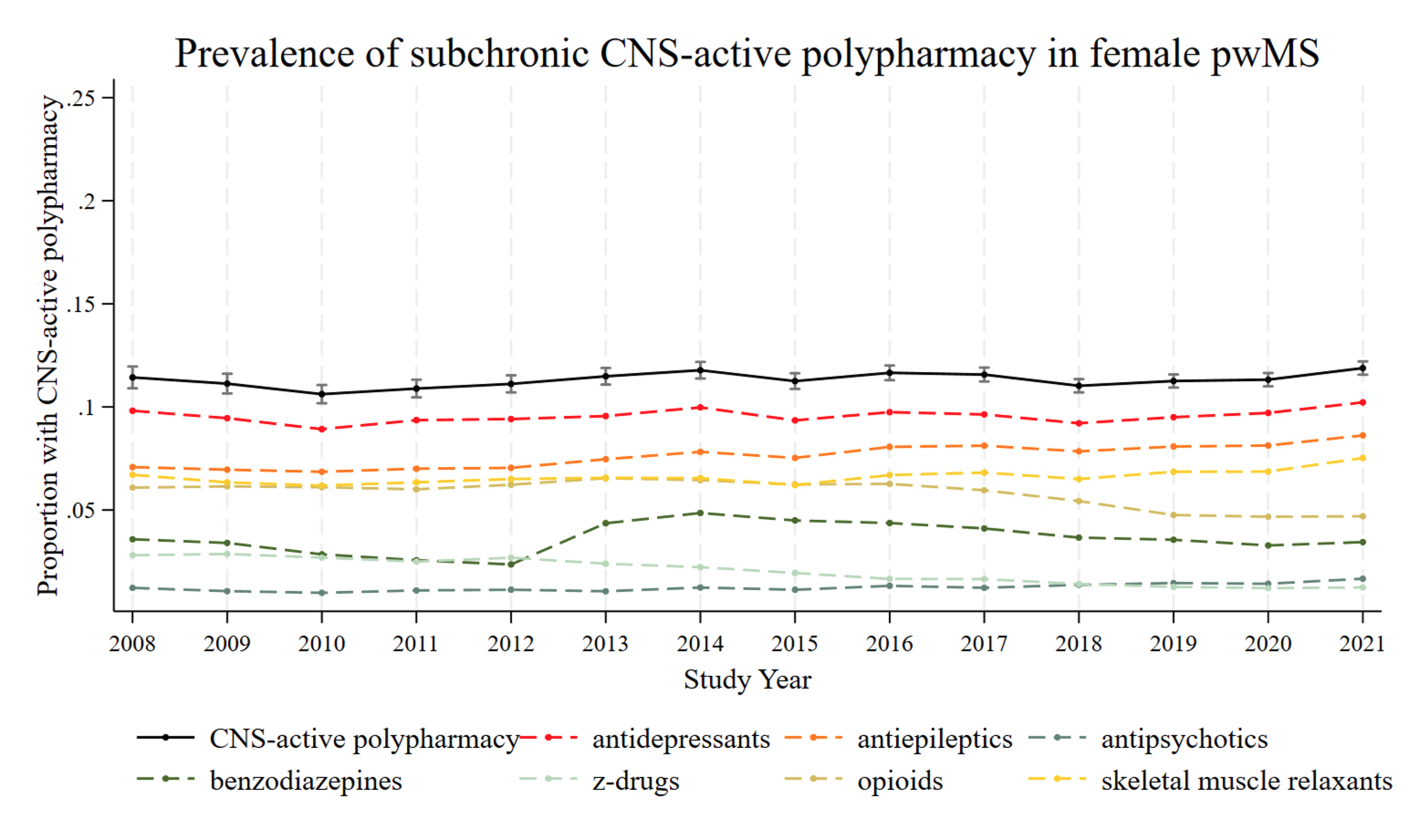 | 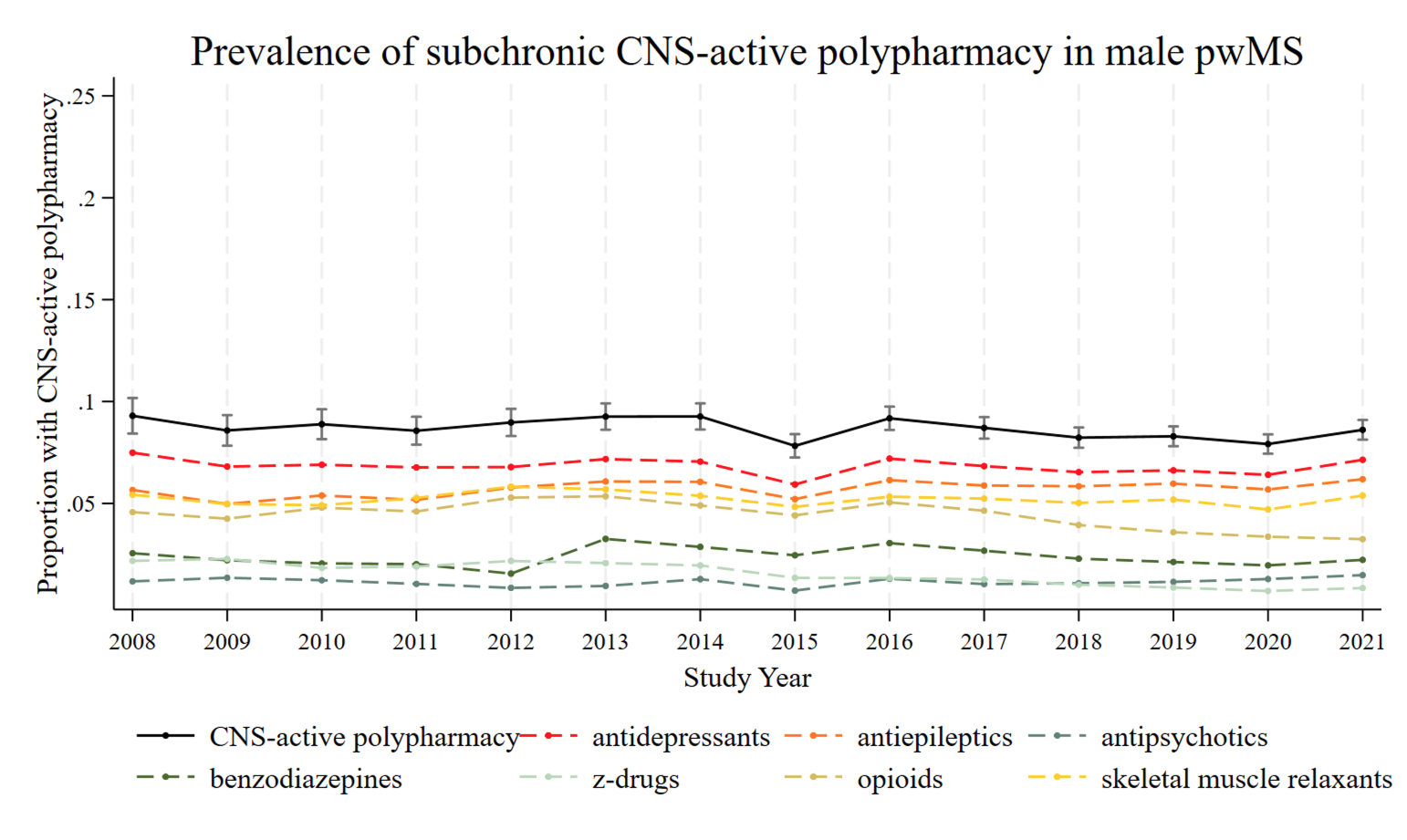 |
|  | 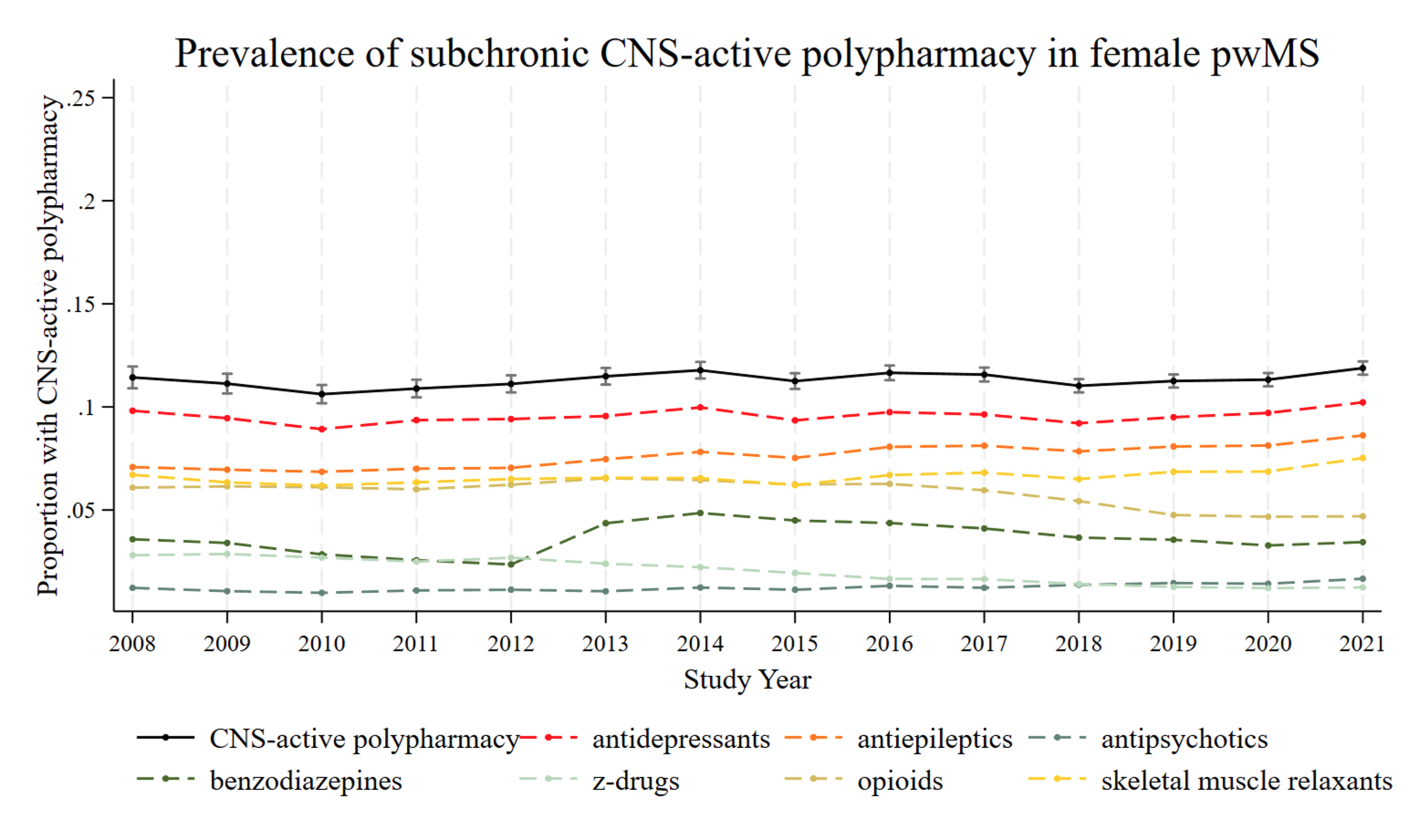 | |

Footnote: 95% CI for not shown for estimates of drug class prevalence

# eTable 4. Descriptive statistics of people with multiple sclerosis in 2021 by sex and CNS-active polypharmacy chronicity

|  | Female PwMS | | | |  |  | Male PwMS | | | |  |
| --- | --- | --- | --- | --- | --- | --- | --- | --- | --- | --- | --- |
|  | Chronic  CNS-active polypharmacy^a^ (n = 6,137) | | Subchronic  CNS-active polypharmacy (n = 4,643) | |  |  | Chronic  CNS-active polypharmacy^a^ (n = 1,351) | | Subchronic  CNS-active polypharmacy (n = 1,079) | |  |
|  | n | (%) | n | (%) | PR^g^ (95% CI) |  | n | (%) | n | (%) | PR^g^ (95% CI) |
| Age |  |  |  |  |  |  |  |  |  |  |  |
| 18 – 35 years | 84 | (1.4) | 129 | (2.8) | **0.49 (0.38–0.65)** |  | 16 | (1.2) | 31 | (2.9) | **0.41 (0.23–0.75)** |
| 36 – 45 years | 544 | (8.9) | 533 | (11.5) | **0.77 (0.69–0.86)** |  | 134 | (9.9) | 129 | (12.0) | 0.83 (0.66–1.04) |
| 46 – 55 years | 1465 | (23.9) | 990 | (21.3) | **1.12 (1.04–1.20)** |  | 305 | (22.6) | 260 | (24.1) | 0.94 (0.81–1.08) |
| 56 – 65 years | 2159 | (35.2) | 1543 | (33.2) | 1.06 (1.00–1.12) |  | 556 | (41.2) | 367 | (34.0) | **1.21 (1.09–1.34)** |
| 66 – 75 years | 1535 | (25.0) | 1128 | (24.3) | 1.03 (0.96–1.10) |  | 293 | (21.7) | 223 | (20.7) | 1.05 (0.90–1.22) |
| ≥76 years | 350 | (5.7) | 320 | (6.9) | **0.83 (0.71–0.96)** |  | 47 | (3.5) | 69 | (6.4) | **0.54 (0.38–0.78)** |
| US Census Region |  |  |  |  |  |  |  |  |  |  |  |
| Midwest | 1313 | (21.4) | 1015 | (21.9) | 0.98 (0.91–1.05) |  | 295 | (21.8) | 232 | (21.5) | 1.02 (0.87–1.18) |
| Northeast | 840 | (13.7) | 571 | (12.3) | 1.11 (1.00–1.22) |  | 193 | (14.3) | 137 | (12.7) | 1.12 (0.92–1.37) |
| South | 2717 | (44.3) | 2065 | (44.5) | 1.00 (0.96–1.04) |  | 523 | (38.7) | 472 | (43.7) | **0.89. (0.81–0.98)** |
| West | 1250 | (20.4) | 981 | (21.1) | 0.96 (0.89–1.03) |  | 336 | (24,9) | 238 | (22.1) | 1.13 (0.97–1.30) |
| Rurality^b^ |  |  |  |  |  |  |  |  |  |  |  |
| Rural | 415 | (6.8) | 280 | (6.0) | 1.12 (0.97–1.30) |  | 71 | (5.3) | 76 | (7.0) | 0.75 (0.55–1.03) |
| Nonrural | 5705 | (93.0) | 4352 | (93.7) | 0.99 (0.98–1.00) |  | 1276 | (94.5) | 1003 | (93.0) | 1.02 (0.99–1.04) |
| Had DMT | 2783 | (45.4) | 1807 | (38.9) | **1.20 (1.14–1.25)** |  | 591 | (43.8) | 465 | (43.1) | 1.05 (0.96–1.14) |
| DMT efficacy |  |  |  |  |  |  |  |  |  |  |  |
| High | 1012 | (16.5) | 684 | (14.7) | **1.20 (1.10–1.31)** |  | 243 | (18.0) | 226 | (21.0) | 0.91 (0.78–1.07) |
| Moderate | 1047 | (17.1) | 666 | (14.3) | **1.22 (1.11–1.33)** |  | 211 | (15.6) | 145 | (13.4) | 1.18 (0.97–1.44) |
| Platform | 724 | (11.8) | 457 | (9.8) | **1.19 (1.07–1.33)** |  | 137 | (10.1) | 94 | (8.7) | 1.17 (0.91–1.50) |
| Had MS relapse^c^ | 729 | (11.9) | 493 | (10.6) | **1.13 (1.02–1.26)** |  | 160 | (11.8) | 137 | (12.7) | 0.93 (0.75–1.16) |
| DME Use | 750 | (12.2) | 488 | (10.5) | **1.16 (1.04–1.29)** |  | 237 | (17.5) | 161 | (14.9) | 1.17 (0.98–1.41) |
| Had MRI | 1958 | (31.9) | 1410 | (30.4) | **1.07 (1.01–1.13)** |  | 397 | (29.4) | 298 | (27.6) | 1.08 (0.95–1.22) |
| Comorbidities, CCI^d^ |  |  |  |  |  |  |  |  |  |  |  |
| None | 4693 | (76.5) | 3740 | (80.6) | **0.95 (0.93–0.97)** |  | 980 | (72.5) | 827 | (76.7) | 0.95 (0.91–1.00) |
| Mild | 1388 | (22.6) | 847 | (18.2) | **1.23 (1.14–1.33)** |  | 340 | (25.2) | 230 | (21.3) | **1.18 (1.02–1.37)** |
| Moderate | 48 | (0.8) | 50 | (1.1) | 0.72 (0.49–1.07) |  | 21 | (1.5) | 15 | (1.4) | 1.12 (0.58–2.16) |
| Severe | 8 | (0.1) | 6 | (0.1) | 1.01 (0.35–2.89) |  | 10 | (0.7) | 7 | (0.7) | 1.14 (0.44–2.97) |
| CNS-related comorbidity |  |  |  |  |  |  |  |  |  |  |  |
| Any CNS comorbidity | 4651 | (75.8) | 3107 | (66.9) | **1.13 (1.10–1.16)** |  | 988 | (73.1) | 707 | (65.5) | **1.12 (1.05–1.18)** |
| Anxiety | 1879 | (30.6) | 1209 | (26.0) | **1.18 (1.11–1.26)** |  | 345 | (25.5) | 234 | (21.7) | **1.18 (1.03–1.37)** |
| Bipolar disorder | 394 | (6.4) | 209 | (4.5) | **1.46 (1.24–1.71)** |  | 75 | (5.6) | 37 | (3.4) | **1.66 (1.13–2.44)** |
| Cognitive impairment | 518 | (8.4) | 354 | (7.6) | 1.10 (0.97–1.25) |  | 141 | (10.4) | 109 | (10.1) | 1.03 (0.82–1.31) |
| Depression | 2606 | (42.5) | 1547 | (33.3) | **1.27 (1.21–1.34)** |  | 526 | (38.9) | 347 | (32.2) | **1.21 (1.08–1.35)** |
| Epilepsy/seizures | 546 | (8.9) | 285 | (6.1) | **1.45 (1.26–1.66)** |  | 126 | (9.3) | 54 | (5.0) | **1.87 (1.38–2.55)** |
| Fatigue/malaise | 1527 | (24.9) | 1069 | (23.0) | **1.08 (1.00–1.15)** |  | 344 | (25.5) | 268 | (24.8) | 1.02 (0.89–1.17) |
| Falls | 205 | (3.3) | 154 | (3.3) | 1.00 (0.82–1.23) |  | 37 | (2.7) | 36 | (3.3) | 0.85 (0.54–1.33) |
| Insomnia | 744 | (12.1) | 432 | (9.3) | **1.30 (1.16–1.45)** |  | 172 | (12.7) | 107 | (9.9) | **1.28 (1.02–1.61)** |
| Malignancy | 375 | (6.1) | 306 | (6.6) | 0.92 (0.80–1.07) |  | 101 | (7.5) | 78 | (7.2) | 1.08 (0.82–1.43) |
| Cancer pain | 14 | (0.2) | 26 | (0.6) | **0.41 (0.21–0.78)** |  | 8 | (0.6) | 5 | (0.5) | 1.31 (0.43–3.95) |
| Noncancer pain | 1577 | (25.7) | 1013 | (21.8) | **1.18 (1.11–1.27)** |  | 267 | (19.8) | 194 | (18.0) | 1.10 (0.93–1.30) |
| Schizophrenia | 114 | (1.9) | 49 | (1.1) | **1.77 (1.27–2.46)** |  | 39 | (2.9) | 22 | (2.0) | 1.45 (0.87–2.43) |
| Somnolence^e^ | 99 | (1.6) | 72 | (1.6) | 1.04 (0.77–1.40) |  | 22 | (1.6) | 20 | (1.9) | 0.88 (0.48–1.60) |
| Alcohol use disorder | 86 | (1.4) | 75 | (1.6) | 0.89 (0.65–1.21) |  | 37 | (2.7) | 39 | (3.6) | 0.77 (0.50–1.20) |
| Non-alcohol use disorder | 382 | (6.2) | 206 | (4.4) | **1.42 (1.20–1.67)** |  | 144 | (8.4) | 61 | (5.7) | **1.54 (1.14–2.08)** |
| Other psychotic disorders^f^ | 82 | (1.3) | 42 | (0.9) | **1.47 (1.01–2.13)** |  | 18 | (1.3) | 16 | (1.5) | 0.90 (0.46–1.75) |

Footnote: Bold indicates statistical significance of prevalence ratio. a – The concurrent use of ≥3 CNS-active drugs with at least >30 days of contiguous exposure; b – rurality defined by zip code classifications from Rural-Urban Commuting Area Codes from the U.S. Department of Agriculture. Metropolitan and micropolitan areas were categorized as nonrural while small town and rural areas were categorized as rural; c – MS relapse, or the acute worsening of neurologic function due to increased MS disease activity, was detected using MS-related inpatient, outpatient, and pharmacy claims data. d – Charlson Comorbidity Index score of 0 indicated no comorbidities, 1 to 2 indicated mild comorbidities, 3 to 4 indicated moderate comorbidities, and ≥5 indicated severe comorbidities; e – includes diagnosis codes for somnolence, stupor, and coma; f – Other psychotic disorders included schizotypal, delusional, brief psychotic, shared psychotic, and other or unspecified psychotic disorders. g – The age-adjusted prevalence ratio of the respective variable by CNS-active polypharmacy status. Prevalence ratios are not age adjusted for the age variable.

# eTable 5. Twenty-five most prescribed CNS-active drugs contributing to subchronic and chronic CNS-active polypharmacy among people with multiple sclerosis in 2021.

| Chronic  Rank | Drug | Prevalence of drug^a^ | | | |  | | Drug class |
| --- | --- | --- | --- | --- | --- | --- | --- | --- |
|  |  | Chronic  CNS-active Polypharmacy^b^  (n = 7,488) | | Subchronic  CNS-active Polypharmacy^c^  (n=5,722) | | Chronic-to-subchronic drug prevalence ratio | |  |
|  |  | n | (%) | n | (%) | Adj. PR^d^ | 95% CI |  |
| 1 | Gabapentin | 4356 | (58.2) | 2795 | (48.8) | 1.19 | (1.15-1.23) | Antiepileptic |
| 2 | Baclofen | 3618 | (48.3) | 2236 | (39.1) | 1.24 | (1.19-1.29) | SMR |
| 3 | Hydrocodone | 2029 | (27.1) | 970 | (17.0) | 1.60 | (1.49-1.71) | Opioid |
| 4 | Duloxetine | 1980 | (26.4) | 1273 | (22.2) | 1.19 | (1.12-1.26) | Antidepressant |
| 5 | Tizanidine | 1915 | (25.6) | 1034 | (18.1) | 1.43 | (1.34-1.53) | SMR |
| 6 | Trazodone | 1885 | (25.2) | 1053 | (18.4) | 1.37 | (1.28-1.46) | Antidepressant |
| 7 | Oxycodone | 1728 | (23.1) | 785 | (13.7) | 1.69 | (1.56-1.83) | Opioid |
| 8 | Bupropion | 1513 | (20.2) | 868 | (15.2) | 1.34 | (1.24-1.44) | Antidepressant |
| 9 | Tramadol | 1160 | (15.5) | 619 | (10.8) | 1.42 | (1.30-1.56) | Opioid |
| 10 | Alprazolam | 1146 | (15.3) | 697 | (12.2) | 1.26 | (1.15-1.37) | Benzodiazepine |
| 11 | Pregabalin | 1114 | (14.9) | 665 | (11.6) | 1.29 | (1.18-1.41) | Antiepileptic |
| 12 | Sertraline | 1073 | (14.3) | 776 | (13.6) | 1.05 | (0.97-1.15) | Antidepressant |
| 13 | Cyclobenzaprine | 989 | (13.2) | 678 | (11.8) | 1.13 | (1.03-1.24) | SMR |
| 14 | Escitalopram | 965 | (12.9) | 702 | (12.3) | 1.05 | (0.96-1.15) | Antidepressant |
| 15 | Topiramate | 918 | (12.3) | 495 | (8.7) | 1.45 | (1.31-1.61) | Antiepileptic |
| 16 | Diazepam | 876 | (11.7) | 450 | (7.9) | 1.50 | (1.35-1.67) | Benzodiazepine |
| 17 | Zolpidem | 831 | (11.1) | 497 | (8.7) | 1.29 | (1.16-1.43) | Z-drug |
| 18 | Venlafaxine | 822 | (11.0) | 488 | (8.5) | 1.29 | (1.16-1.43) | Antidepressant |
| 19 | Lorazepam | 818 | (10.9) | 424 | (7.4) | 1.47 | (1.31-1.64) | Benzodiazepine |
| 20 | Fluoxetine | 748 | (10.0) | 502 | (8.8) | 1.14 | (1.02-1.27) | Antidepressant |
| 21 | Amitriptyline | 737 | (9.8) | 474 | (8.3) | 1.20 | (1.07-1.34) | Antidepressant |
| 22 | Quetiapine | 724 | (9.7) | 363 | (6.3) | 1.53 | (1.36-1.73) | Antipsychotic |
| 23 | Lamotrigine | 646 | (8.6) | 309 | (5.4) | 1.43 | (1.26-1.62) | Antiepileptic |
| 24 | Mirtazapine | 640 | (8.5) | 342 | (6.0) | 1.14 | (1.02-1.29) | Antidepressant |
| 25 | Citalopram | 632 | (8.4) | 419 | (7.3) | 1.19 | (1.15-1.23) | Benzodiazepine |
| Footnote: Definitions: SMR = Skeletal muscle relaxant; a- number of pwMS that had a prescription for the respective CNS-active drug during any period of CNS-active polypharmacy; b – age-adjusted prevalence of the concurrent use of ≥3 CNS-active drugs with at least >30 days of contiguous exposure for ≥180 days c – age-adjusted prevalence of the concurrent use of ≥3 CNS-active drugs with >30 days to <180 days; d – age and sex-adjusted drug prevalence ratio between pwMS with chronic CNS-active polypharmacy versus subchronic CNS-active polypharmacy. | | | | | | | | |
|  | | | | | | | | |

# eTable 6: Count and proportion of prescription-days contributing to CNS-active polypharmacy in people with multiple sclerosis in 2021 by CNS-active drug class in the five most common prescriber types

| Rank |  | Antidepressants  (n = 1,770,148) | | Antiepileptics  (n = 1,274,512) | | Antipsychotics  (n = 215,379) | | Benzodiazepines  (n = 224,447) | | Z-drugs  (n = 96,958) | | Opioids  (n = 372,402) | | Skeletal Muscle Relaxants  (n = 818,187) | | Overall  (n = 4,772,033) | |
| --- | --- | --- | --- | --- | --- | --- | --- | --- | --- | --- | --- | --- | --- | --- | --- | --- | --- |
|  | Prescriber Type | n | % | n | % | n | % | n | % | n | % | n | % | n | % | n | % |
| 1 | Primary care physician | 830630 | **46.9** | 399653 | 31.4 | 71593 | **33.2** | 97303 | **43.4** | 45939 | **47.4** | 124207 | **33.4** | 251080 | 30.7 | 1820405 | 38.2 |
| 2 | Neurologist | 275867 | 15.6 | 459037 | **36.0** | 10654 | 5.0 | 43311 | 19.3 | 17960 | 18.5 | 28891 | 7.8 | 318659 | **39.0** | 1154379 | 24.2 |
| 3 | Non-physician practitioner | 204872 | 11.6 | 138390 | 10.9 | 29609 | 13.8 | 24945 | 11.1 | 11047 | 11.4 | 53794 | 14.5 | 99018 | 12.1 | 561.675 | 11.8 |
| 4 | Psychiatrist | 164431 | 9.3 | 50297 | 4.0 | 54666 | 25.4 | 23403 | 10.4 | 7899 | 8.2 | 2798 | 0.8 | 5571 | 0.7 | 309065 | 6.5 |
| 5 | Unknown | 90808 | 5.1 | 65458 | 5.1 | 12114 | 5.6 | 11119 | 5.0 | 3138 | 3.2 | 21887 | 5.9 | 39003 | 4.8 | 243527 | 5.1 |
| Footnote: Bold indicates provider type with highest proportion of prescriptions by drug class. | | | | | | | | | | | | | | | | | |
